# Ethical and legal aspects of patient’s safety: a clinical case report

**Published:** 2017-12-30

**Authors:** Maliheh Kadivar, Arpi Manookian, Fariba Asghari, Nikoo Niknafs, Arash Okazi, Asal Zarvani

**Affiliations:** 1 *Professor, Division of Neonatology, Department of Pediatrics, Children’s Medical Center, Tehran University of Medical Sciences, Tehran, Iran. *; 2 *Assistant Professor, School of Nursing & Midwifery, Tehran University of Medical Sciences, Tehran, Iran. *; 3 *Associate Professor, Medical Ethic* *s* * and History of Medicine Research Center, Tehran University of Medical Sciences, Tehran, Iran. *; 4 *Assistant Professor, Division of Neonatology, Department of Pediatrics, Vali-e-Asr Hospital, Tehran University of Medical Sciences, Tehran, Iran. *; 5 *Assistant Professor, Department of Forensic Medicine, Tehran University of Medical Sciences, Tehran, Iran.*; 6 *Nursing Student, School of Nursing & Midwifery, Tehran University of Medical Sciences, Tehran, Iran. *

**Keywords:** *Patient safety*, *Risk management*, *Ethics*, *Legislation*, *Case study*

## Abstract

Since patient safety is multidimensional and grounded in ethical and legal imperatives, both ethical and legal challenges should be taken into account. In this regard, a falling incident case of a 12-day-old newborn was raised in the monthly ethics round in the Children's Medical Center of Tehran University of Medical Sciences, Iran, and the ethical and legal dimensions of patient safety were discussed by experts in various fields.

This report presents different aspects of patient safety in terms of root cause analysis (RCA) and risk management, the role of human resources, the role of professionalism, the necessity of informing the parents (disclosure of medical errors), and forensic medicine with focus on ethical aspects.

## Introduction

According to the non-maleficence principle of medical ethics, ensuring patients’ safety and preventing any injury or damage to them is a major priority for healthcare providers (1). Thus, it has been the most emphasized component of the quality of health care services all around the world. The Institute of Medicine (IOM) released a report in 1999 entitled "Man is fallible: create a safe health system" in relation to the incidence of medical errors in United States, and consequently, initiated widespread international change in the field of patient safety (2). Correspondingly, the Iranian health care system implemented special plans purposed to deliver standard health care services and prevent any mistakes and an organized approach to risk management, systematic deficiency, and patient safety improvement (3). One of these programs is clinical governance which was introduced by the Ministry of Health and Medical Education (MOHME) and initiated since November 2009. Although great emphasis has been placed on the importance of clinical governance by the MOHME, there are some challenges in achieving the desired outcomes (4). This could be the result of healthcare providers’ inadequate understanding about the importance of clinical governance and lack of organizational safety culture (5, 6). 

Studies showed that a non-negligible percentage of patients are exposed to health care-related injuries. Based on World Health Organization (WHO) report, the possibility of harming patients in the process of providing health care services is 1 out of 300, whereas the possibility of aviation accidents is 1 out of 100,000. Since 2004, with the beginning of the patient safety project, so far 140 countries have attempted to improve their patients' safety plans in their own health system (7). The most common cause of injury is medication errors and falling. Although falling includes 21% of total incidents, only 4% of them are serious. Meanwhile the neonatal falling statistics in the USA is 1.6-4.4 in 10,000 live births, an estimated 600-1600 falling incidents in a year. These cases are often the result of shortcomings in systems and processes, organizational complexity and ambiguity, and poor communication (8, 9).

Despite various patient safety guidelines and standards, less attention is paid to the ethical and legal aspects of this issue. From a moral perspective, the main goal of patient safety in the health system can be studied from two aspects. It can be studied as a practical value, in the sense that the main focus is its positive outcomes and benefits. It can also be studied as a moral value by focusing on the protection and promotion of humanity and human dignity. It should be emphasized that both aspects are important in the health system. From a professional point of view, moral values in patient safety are not separated from basic medical obligations, but are so central that they may be the source of other moral values emphasized in medicine. This means that patient safety is closely related to the concept of human dignity and all patient safety measures taken must insure the protection of human’s dignity (10). In other words, the responsibility of the health care staff and professional commitment, in general, are closely related to human dignity (11).

This case was raised in the monthly ethics round in the Children's Medical Center of Tehran University of Medical Sciences, Iran, and ethical dimensions of patient safety were discussed by experts in various fields. The opinions expressed in this article are a summary of the views of experts in various fields including neonatology, medical law, ethics, and nursing.

It is worth mentioning that the ethics round has been held every month for more than 5 years in the Children's Medical Center. A complicated case is discussed in each session with the presence of different relevant experts.

**The clinical case**

A 12-day old newborn infant was hospitalized in the neonatal intensive care unit (NICU) because of multiple seizures. He was the first child of the family. The mother’s and family history was negative for seizures or any other disease. Seizures were controlled by medications, and diagnostic assessments including electroencephalography (EEG) were ordered. As the infant was stable and had tolerated breast feeding, it was planned to transfer him to the level II NICU, but it was postponed due to lack of available beds.

On the evening shift of the third day of admission, his nurse heard a sudden noise and noticed that the incubator door was open and the baby was on the floor. The in-charge nurse immediately announced the incident to the on-call physician. The newborn was examined thoroughly and no physical injuries were found. Moreover, the incident was reported to the chief physician of the department and the record of this incident was immediately sent to the hospital officials. Later, all other incubators were inspected to make sure they were secure enough. 

When the staff informed the newborn’s father of the falling, he accused the mother of neglecting the child, although she had said that she was resting at the time of the incident.

The questions raised in the meeting were as follows: 

What are the factors leading to this incident? How could this incident been prevented? What is the responsibility of the staff in dealing with this incident? Based on professional commitments, what is the duty of medical and nursing staff in such events? What are the ethical issues of patient safety in this case? What are the legal obligations and consequences of this case?

## Discussion

**Root cause analysis and risk management**

Searching for the causes and finding the right solution, in other words, the basic analysis of the incident is one of the initial and essential measures taken to decrease the incidence of patient injuries. It should be noted that the mentioned process must be free of any bias and should focus on finding the main cause and resolving it instead of identifying the responsible person. One way of preventing such events is to have a special guideline for reporting the event in a suitable organized ethical atmosphere without accusing anyone. Indeed, fear of blame, penalties, limited organizational support, inadequate feedback, and lack of knowledge about the associated factors are some of the barriers to reporting medical errors in hospitals (12). 

Assessment and reduction of patients’ risk of injury, or risk management in the clinical setting is influenced by several factors. One way is the establishment of an organizational culture based on mutual trust and effective communication in all hospital levels (13, 14). 

From an ethical perspective, the value of trustworthiness is a prerequisite of successful risk management. This value is connected to safety culture since it refers to physical safety, psychological safety, and cultural safety. Thus, the managers’ responsibility is to create mental and physical safety settings based on openness in order to promote patient safety and care quality. Furthermore, it is important for the managers to encourage multidisciplinary collaboration to facilitate transparent reporting (10).

In this case, the apparent reason was that the incubator door was left open by someone or was not correctly closed. Questions raised in this context include the following: Was the nurse occupied with other emergency and essential actions? Was the incubator door latch broken? Why would the nurse forget to accurately examine the door? Is it possible that lack of guidelines for patient safety led to this incident? 

The most important step to reduce the possibility of such events in clinical settings is to establish policies and procedures that work best for each ward. Furthermore, the continuous training of the personnel in patient safety, steady supervision, and controlling the efficacy level of the performed actions are some other steps that can be taken in this regard. For instance, in this case, frequent checking of the incubator door, the use of two locks, and explanation of safety tips regarding the incubator to the staff are also important. Furthermore, evaluation and constant controlling of compliance with patient safety rules, and feedback are also necessary.

**The role of human resources**

The quantity of human resources is also noteworthy in the field of patient safety. In other words, quality assurance depends on the quantity of manpower. Therefore, in order to prevent similar incidents, providing an adequate number of staff at the bedside is essential (12).

**The role of professionalism**

Professional ethics and patient safety are intertwined fundamental concepts in medicine. Patient safety is grounded in ethical principles which are considered as care quality indicators (15). The realization of patient safety requires the provision and implementation of a professional code of ethics. Based on the Iranian healthcare professional code of conduct, it is expected that all patients be treated with dignity and be protected from any possible harm (16). Accordingly, adherence to ethical principles requires healthcare providers to identify potential safety failures to prevent falling incidents (15). 

The establishment of patient safety has different individual, professional, and organizational aspects with a special focus on ethics. Professional and organizational commitment leads to detecting and reporting of both one’s own and others’ errors (10). 

From an ethical view, the following actions are recommended:

Following professional and institutional guidelines (if any exist) related to falling incidents; Taking basic actions to assess the patient’s physical health and rescue his/her life; Informing the in-charge staff; Punctual assessment of the situation, and complete documentation and reporting of the event (important data such as the time of the incident, the infant’s position, level of consciousness, vital signs, those present at the scene, actions taken in the process, and etc.) should be documented; Informing the parents and providing them with emotional support.

**Informing parents (Disclosure of medical errors)**

It seems in case of any error made by the care team, the event must be announced to the parents honestly without blaming the care providers. Moreover, irritating phrases such as “It happens” and “Nothing has happened though” should not be used. 

Under circumstances in which errors were caused by inappropriate pattern of providing hospital services, parents should be reassured that all services will be paid for by the hospital. It would be better if the parents were informed by the chief physician or the head nurse and given enough time to express their concern or anger.

Although anger under such circumstances is a natural reaction, we cannot hide medical errors because of fear of parents’ reaction. Moreover, parents’ anger would be more severe if they found out that the hospital personnel have concealed the truth. 

It should be considered that knowing the truth is one of the basic rights of patients and their family members. According to similar studies, explaining the error to the patients could be a stressful situation combined with intense emotional reactions from patient/family members or the care team. Generally, the person who committed the error has a sense of guilt or fear of punishment, and patient/family members experience feelings such as anger and anxiety. Furthermore, it should be noticed that primary conversations usually take place when there is not accurate and comprehensive information about the event, so recognizing, understanding, and explaining all the details in complicated clinical situations is not possible. Thus, it is suggested that in such situations, information be given in several stages and by providing psychological support for the patient. Furthermore, while they may need supportive interventions, the patient's family can be considered as an important source of information in the process of root cause analysis (RCA) of similar incidents (17-20). Since creating ethical patient safety is a multidimensional accomplishment, it should be considered that actively partnering with the patient’s family may be a high-yield approach to detecting and preventing medical errors (10).

In addition, regarding the presented case, the father should be ensured that hospitalizing the newborn was necessary and the mother should not be blamed. In fact, he should be ensured that the incident was entirely due to system error and not by the mother. Basically, maintaining the integrity of the family is essential and medical staff must consider family support at all stages, especially in such circumstances.

Indeed, an important ethical point in this case is the necessity of offering an honest apology. It is not always as simple as saying: “We are sorry”. The way of informing the parents is a sensitive issue and there is an urgent need for training healthcare providers in sensitive interpersonal relationships and related skills to facilitate honest and proper communication with the patient’s family (20).

**Forensic medicine aspect**

Laws and regulations related to patient safety, which may vary based on the legislation system of each country, should encourage the disclosure of medical errors while supporting the implementation of the ethical imperatives of patient safety. In general, based on the medical law, the patient who is a victim of negligence is supposed to be fairly compensated. In addition, these rules provide possibilities for promotion of transparency and open communications in all levels. Reaching this goal requires regarding all stakeholders in the healthcare system (21). 

In the mentioned case, some questions could be raised. Either the falling was in the presence of the mother or not. If it was in her presence, the hypothesis is that she dropped the baby intentionally. However, if there is no sign of any apparent trauma, it seems there was no specific hurtful force or he fell from his mother’s arms, and it shows the mother’s lack of experience. 

Accordingly, it should be considered that maternal postpartum sleepiness is one of the major risk factors for falling of newborns. Half of all newborn falling incidents in hospitals have occurred while the mother was holding the infant in a hospital bed. Therefore, recognizing the risks of neonatal falling during mother–baby care situations and teaching the mothers is a major nursing responsibility (22).

If the mother is incapable of taking care of the hospitalized baby, she should be under supervision of the care team and should be educated. Furthermore, notifying the father is an appropriate act if the complaint was raised by the father.

The main task of the physician or nurse after a detailed examination and treatment is the detailed registration and description of all events without any assumptions. If the examinations found evidence of neglect, it would be a completely different discussion and calling the social services would be absolutely necessary. 

The question might also be raised that “if nothing happened to the child and we did all the assessments to insure his health, are we obligated to inform the parents?”

There is an obligation to inform the patient or the family about every unwanted event in healthcare settings. The idea that there is no need to disclose errors which did not affect the patient is based on the traditional stance of the law. Furthermore, today, it is well known that such disclosures will enhance patients’ trust to healthcare professionals while making them aware of that which is going on around them. In addition, through this approach, healthcare professionals can respect the patients’ autonomy and dignity (21, 23). 

## Conclusion

Despite increased attention toward the quality of health care services, there are still numerous threats to patient safety in healthcare settings. Since patient safety is multidimensional and grounded in ethical and legal imperatives, both ethical and legal challenges should be taken into account. 

Reaching the ultimate goal of the healthcare system, which is to ensure quality and safety of the services, requires structured policies and processes to foster the safety settings based on mutual trust. This can be facilitated by encouraging multidisciplinary collaboration for the transparent reporting of medical errors and also active participation of the patients and their families in detecting medical errors. Furthermore, the provision of emotional support and legal protection of the staffs by the organization is essential to encourage voluntary reporting of incidents. 

Moreover, training and emphasizing on the professional code of ethics can be effective on deepening the understanding of and belief in the moral foundations of patient safety.
